# Regulation of actin nucleation and autophagosome formation

**DOI:** 10.1007/s00018-016-2224-z

**Published:** 2016-05-04

**Authors:** Amanda S. Coutts, Nicholas B. La Thangue

**Affiliations:** Laboratory of Cancer Biology, Medical Sciences Division, Department of Oncology, University of Oxford, Old Road Campus Research Building, Old Road Campus, Off Roosevelt Drive, Oxford, OX3 7DQ UK

**Keywords:** Autophagy, Actin, Autophagosome, JMY, Arp2/3, WH2, LC3

## Abstract

Autophagy is a process of self-eating, whereby cytosolic constituents are enclosed by a double-membrane vesicle before delivery to the lysosome for degradation. This is an important process which allows for recycling of nutrients and cellular components and thus plays a critical role in normal cellular homeostasis as well as cell survival during stresses such as starvation or hypoxia. A large number of proteins regulate various stages of autophagy in a complex and still incompletely understood series of events. In this review, we will discuss recent studies which provide a growing body of evidence that actin dynamics and proteins that influence actin nucleation play an important role in the regulation of autophagosome formation and maturation.

## Introduction

**Fig. 1:**
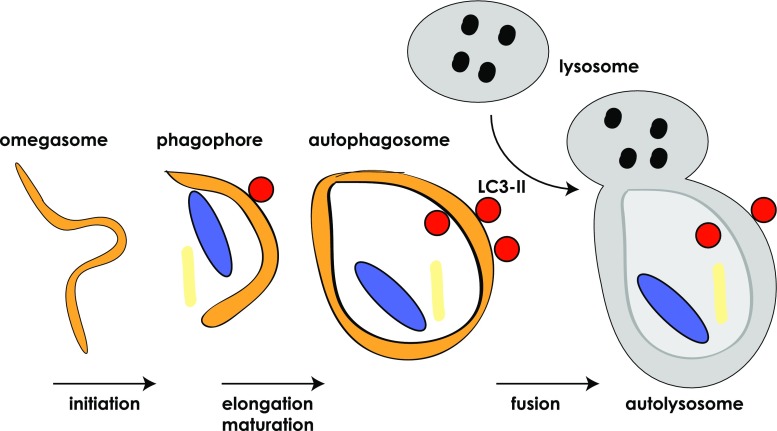
Autophagosome formation. Simplified *cartoon* depicts the stages of autophagosome formation, starting with the initiation at the omegasome until finally the mature autophagosome fuses with the lysosome to become the autolysosome. At this point the lysosome releases its cargo of degradative enzymes into the autophagosome to degrade the contents, including the inner autophagosomal membrane. Lipidated LC3 (*red circles*) associates with the autophagosome and remains there until it is recycled after degradation by the autolysosome

Autophagy is a process of self-eating, whereby cytosolic constituents are enclosed by a double-membrane vesicle before delivery to the lysosome for degradation. This allows for recycling of nutrients and cytoplasmic components and thus plays an important role in normal cellular homeostasis as well as survival during stress and pathophysiological processes such as cancer, infection, neurodegenerative and metabolic disorders [1]. While autophagy is a constitutive process it can be rapidly regulated by a variety of stimuli, for example, nutrient starvation, hypoxia and DNA damage [2, 3]. A large number of proteins orchestrate various stages of autophagosome formation in a complex and highly regulated process that begins with membrane sequestration at the omegasome which forms the double-membraned phagophore (also referred to as isolation membrane) [4]. The phagophore gradually elongates and matures to close in on itself before ultimately fusing with the lysosome to form the mature autophagosome (Fig. 1) [5]. During this process, cytoplasmic components (such as misfolded proteins and damaged organelles) become enclosed by the maturing (expanding) phagophore [6]. In addition, proteins can be specifically targeted to the autophagosome via a chaperone protein, with p62 being the classic example [7]. There have been described a number of types of autophagy (including, macroautophagy, microautophagy and chaperone-mediated autophagy; [8]), but we will hereafter be using autophagy to relate primarily to macroautophagy.

Autophagy was first described morphologically over 50 years ago [9] and although now we know much about the molecular details, the proteins and mechanisms that regulate this complex and fundamental process are still being uncovered. Of particular interest is the fact that recent studies have demonstrated that actin nucleation is involved in the regulation of autophagosome formation and maturation, and in this review we will focus on the experimental evidence for this role and how this exciting new area could aid our understanding of autophagosome formation.

## Autophagy formation

**Fig. 2:**
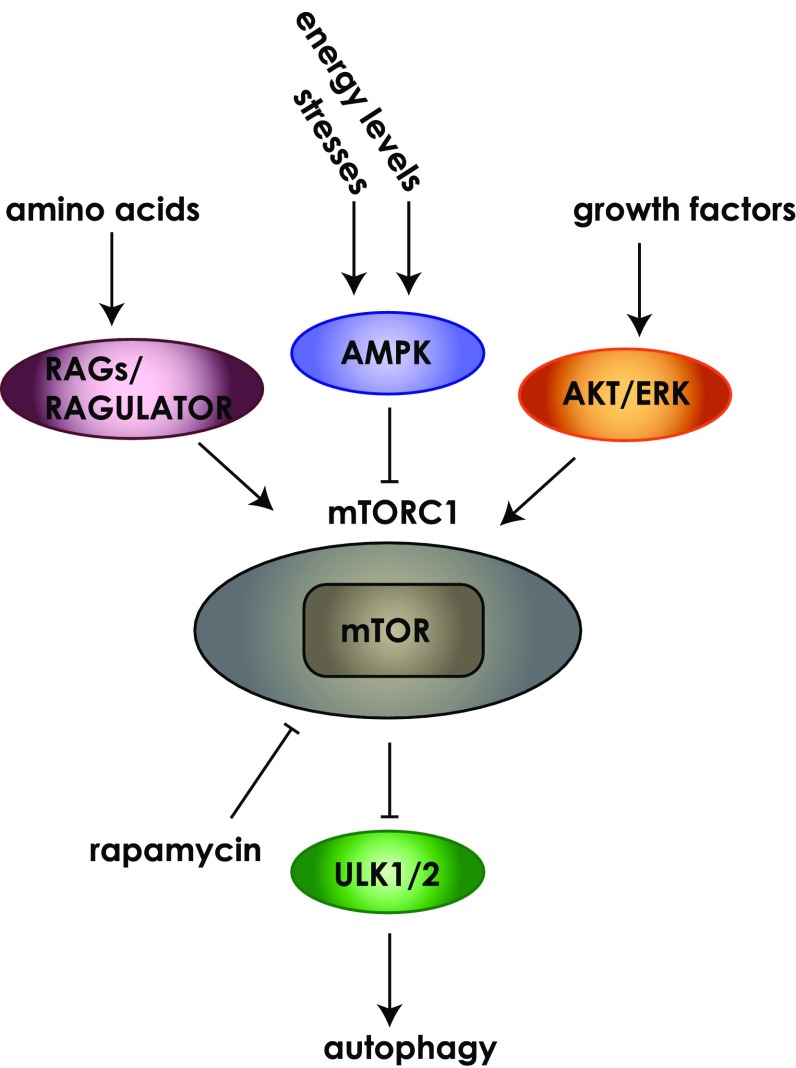
Signalling upstream of mTORC1 regulates autophagy. Many signalling pathways converge on the mTORC1 complex to control its activation state. Examples of these include; energy signals controlled by the AMP/ATP ratio, nutrient signals from growth factors or amino acids, stress signals such as DNA damage and hypoxia. The mTORC1 complex contains the highly conserved serine/threonine kinase mTOR. Rapamycin can inhibit mTOR activity leading to activation of autophagy. Growth factor receptor signalling can activate pathways such as ERK or AKT. Amino acid signalling through RAGs and the RAGULATOR complex and activation of AMPK through sensing of energy levels can also feed into mTORC1 activity to influence autophagy. *Cartoon* does not reflect the relative size of the proteins

Autophagy was first described in yeast cells where approximately 40 autophagy-related genes (Atg) have since been identified [10–12]. Autophagy is a well conserved process and many of the initial observations in yeast have been shown to be relevant to autophagy in mammalian cells [13]. Roughly half of these form the core autophagy proteins that comprise several discrete protein complexes [5, 12]. In addition, many non-Atg proteins are involved in the regulation of, and various steps in the formation of, the autophagosome [14]. Notably, autophagosome formation is unique in comparison to the generation of other intracellular vesicles in that it does not employ a budding process but instead autophagosomes are formed de novo from bending and expansion of the membrane [15]. Autophagosome formation depends on the rapid recruitment of membrane in often large amounts to form the phagophore (also referred to as isolation membrane, [16]). While endoplasmic reticulum (ER) subdomains enriched in phosphatidylinositol-3-phosphate (PI(3)P), called omegasomes, are thought to be a major source of autophagsomal membrane [17, 18], evidence also supports other sources such as the Golgi, mitochondria, ER-mitochondria junction and plasma membrane [4]. The cup-shaped omegasome is a transient structure that can be identified by the presence of DFCP1 (double FYVE domain-containing protein 1) and develops into the expanding phagophore [17]. Many stimuli have been demonstrated to result in autophagy including nutrient and amino acid depletion, and the mTORC1 (mammalian target of rapamycin complex 1) pathway plays a critical role in the regulation of autophagy (Fig. 2). For example, AMP-activated protein kinase (AMPK) is a key energy sensor and when cellular energy levels are low, AMPK is activated leading to phosphorylation of target proteins including RAPTOR and TSC1/2 resulting in mTORC1 inhibition and activation of autophagy [2]. In general, under normal growth conditions, when nutrients are replete, mTORC1 is active and will suppress autophagy, whereas nutrient depletion results in mTORC1 inhibition leading to activation of the autophagocytic process [2].

### Initiation and nucleation

Numerous signalling pathways converge on mTORC1 to control its activation state (Fig. 2) and signals feed into the initiation of autophagosome formation via the ULK (unc-51-like kinases) complex which is composed of the serine-threonine kinases ULK1/2, along with Atg13, Atg101, FIP200 (focal adhesion kinase family-interacting protein 200 kDa) [19]. Nutrient-depleted conditions result in the dissociation of mTORC1 from the ULK complex and stimulate autophagosome formation [19]. Recent studies have identified a number of substrates of ULK1/2 that likely play important roles in the regulation of autophagy initiation, including Atg13 and FIP200, but the molecular details remain to be elucidated [14, 20]. Despite this, it has been shown that the kinase activity of ULK1/2 is necessary for autophagy initiation [21, 22].

The ULK kinase complex is required for the recruitment of the class III phosphatidylinositol-3-kinase (PI(3)K) complex containing Beclin1 (Atg6) [23]. Beclin1 is an important initiator of mammalian autophagy, as it recruits a complex containing the PI(3)K vacuolar protein sorting 34 (Vps34), p150 and Atg14 to the pre-autophagosomal structure [16]. The Beclin complex may also contain other proteins, such as AMBRA1 or UVRAG which can influence its function [24]. The PI(3)K Vps34 phosphorylates phosphatidylinositol at the autophagosomal membrane to generate PI(3)P [16]. Once both the ULK kinase and Beclin complexes are active and at the site of initiation this will lead to phosphorylation of downstream targets, including Beclin1 [23] and the accrual of PI(3)P at the initiation site [16]. Further recruitment of proteins that can influence autophagosome formation, such as WIPI (WD-repeat domain phosphoinositide-interacting protein) and DFCP1 both PI(3)P effectors, is thought to help to drive progression of the omegasome to the isolation membrane [25–27].

Initiation of autophagy also requires Atg9 (mammalian Atg9A) which is recruited to the autophagosome formation site and in yeast this requires an interaction between the Atg1 and Atg13 sub-complex and Atg9 [28, 29]. Of note, Atg9 is the only transmembrane Atg protein identified so far [30, 31] and it has been suggested that Atg9 may be involved in providing membrane for autophagosome formation [32, 33]. Atg9 is localised at the *trans*-Golgi network and late endosomes and during autophagy is trafficked to endosomal membranes [31]. In addition, Atg9 is a target of the Atg1/ULK1 kinase and phosphorylation is thought to be involved in the early stages of autophagosome formation leading to autophagosome expansion [34].

### Expansion

Two ubiquitin-like complexes are involved in autophagosome elongation and maturation. The first, the Atg16L complex, involves the ubiquitin-like Atg12 which conjugates with Atg5 in a process requiring Atg7 and Atg10 [35, 36]. Atg5–Atg12 then interacts with Atg16L and associates with the phagophore [37, 38]. The second involves the lipidation of LC3 (Atg8/microtubule-associated protein 1A/B light chain 3; LC3-I) family members (which include the LC3, GATE16 and GABARAP subfamilies) [14]. Atg4 cleaves pro-LC3 to form LC3-I which is then conjugated to phosphatidylethanolamine (PE) via Atg3–Atg7 and Atg5–Atg12 to form (LC3-II) [39–41]. This allows the association of LC3-II with the autophagosome where it remains bound until it is recycled during lysosomal degradation and is thus considered one of the most reliable markers of autophagy [42].

## Autophagosome closure and fusion with the lysosome

Closure of the autophagosome is a less well understood process, but it is thought that the maturing double-membraned phagophore must undergo some type of closure/scission event before ultimately fusing with the lysosome in order to degrade its cargo [43, 44]. Over-expression of a protease-deficient Atg4B mutant resulted in cells that displayed a defect in autophagosome closure [45]. Since Atg4B processes LC3 this implies that LC3 may be involved in the final stages of autophagosome formation [45]. A similar phenotype was observed in Atg3 knockout cells in which LC3 lipidation is prevented [46]. Together these studies suggest that the cleaved and lipidated form of LC3 (LC3-II) is likely involved in later stages of autophagosome formation, although further work is required to work out the molecular details. Proteins that interact with LC3 including Atg proteins contain a consensus LC3-interacting region (LIR) whose core sequence is [W/F/Y]xx[L/I/V] [47]. It could be that LC3-II recruits proteins involved in the final stages of autophagosome maturation perhaps via LIR interactions to mediate closure of the autophagosome.

Once formed, autophagosomes move rapidly toward lysosomes in the peri-nuclear region of the cell in a microtubule-dependent fashion [48]. When in contact with a lysosome the outer membrane of the autophagosome fuses with the lysosomal membrane, releasing the degradative enzymes into the now mature autolysosome [13]. Fusion relies on the Rab-SNARE system which is more generally involved in processes requiring vesicle fusion [44]. Other autophagy proteins may also play a role in autophagosome fusion, such as UVRAG [49] and Atg14 [50] although the molecular details have yet to be fully worked out. More recent work has shown that the Atg8 orthologues the γ-aminobutyric acid receptor-associated proteins (GABARAPs) are involved in autophagosome to lysosome fusion [51]. Interestingly, GABARAPs recruit the lipid kinase PI(4)KIIα (phosphatidylinositol-4-kinase IIα) to the autophagosome where it generates PI(4)P (phosphatidylinositol-4-phosphate) [51]. PI(4)KIIα and the generation of PI(4)P were shown to be required for fusion of the autophagosome with lysosome, although it is currently unclear how PI(4)P production drives fusion [51].

## Actin nucleation

Autophagosome formation is a complex series of discrete events, mediated and controlled by a large number of proteins. Adding to this complexity is recent work describing roles for actin nucleation promoting proteins in various stages of autophagosome formation, demonstrating a role for actin in autophagosome formation. Actin fibres consist of two antiparallel chains of filamentous actin (F-actin) coiled to form a right-handed helix [52]. F-actin is composed of globular actin (G-actin) subunits that can be assembled and disassembled in a rapid and dynamic fashion, mediated by a wide variety of proteins including polymerisation and depolymerisation factors as well as capping proteins [53]. Actin filaments have an inherent polarity provided by their fast growing barbed (plus) ends and their more slowly growing pointed (minus) ends [54]. The fast growing barbed ends are often pointed towards membranes where associated proteins can signal rapid filament growth to generate force and movement [54].

**Fig. 3:**
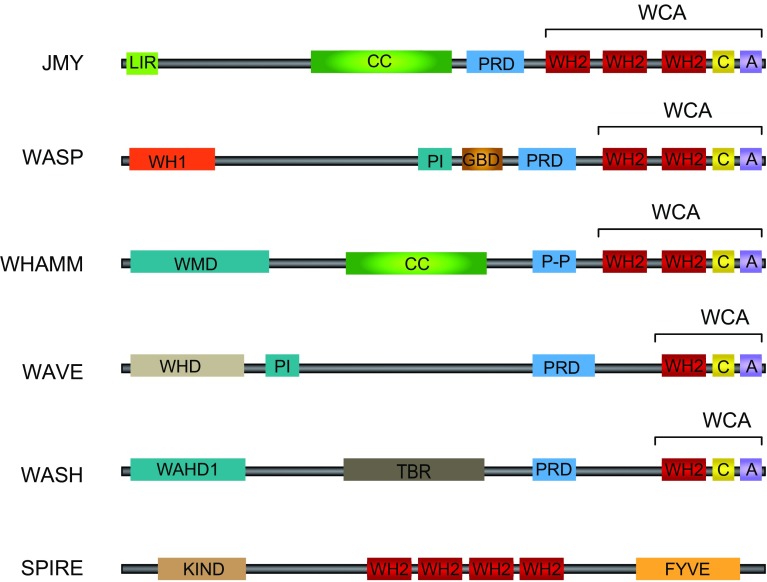
Structural organisation of actin nucleators. Class I NPFs include JMY, WASP, WAVE and contain C-terminal WCA regions containing the actin-binding WH2 domains. The tandem monomer-binding protein class includes JMY and Spire and also contain WH2 domains, but do not require Arp2/3 binding for actin nucleation activity. The N-terminal regions of actin nucleators are often highly divergent and contain binding sites for various regulatory factors including phosphoinositides (PI) and small GTPases (GBD). *A* acidic, *C* central, *CC* coiled-coil, *KIND* kinase non-catalytic C-lobe domain, *LIR* LC3-interacting region, *PRD* proline-rich domain; *P-P* poly-proline, *TBR* tubulin binding region, *WAHD1* WASH homology domain 1, *WHD* WAVE homology domain, *WMD* WHAMM membrane interaction domain. Adapted from [61, 62]

The initial step of actin filament formation, nucleation, occurs when actin monomers first combine into an actin dimer or trimer and this is the rate-limiting step of actin polymerisation [54]. The subsequent addition of further actin monomers to these stable nucleation seeds is energetically favourable and results in rapid filament elongation. A large number of actin-binding proteins are involved in the regulation of actin filament dynamics. In cells spontaneous actin polymerisation is inhibited by proteins that sequester actin monomers (such as profilin and thymosin β4), and nucleation promoting factors (NPFs) are used to overcome this inhibition [52]. These NPFs contain G-actin binding sites, for example WASP-homology 2 (WH2) domains, to bind and sequester actin monomers to form a seed for subsequent nucleation (Fig. 3) [55]. Importantly, the assortment of nucleation factors allows cells to direct actin filament formation in a temporal and spatial manner while also allowing for a variety of actin structures [52].

Actin nucleation factors fall into four major classes: (1) the Arp2/3 (actin-related protein 2/3) (2) the NPFs, (3) the formins and (4) the tandem-monomer-binding nucleators. The Arp2/3 complex is a major regulator of actin filament assembly and the first actin nucleator to be discovered [56, 57]. It is composed of the actin-related proteins Arp2 and Arp3 and five additional highly conserved subunits [58]. The Arp2/3 complex nucleates branched actin filaments from the side of existing filaments [59]. Arp2/3-dependent actin nucleation can generate pushing force against a membrane and in this way allows it to function in various cellular processes such as cell migration, cell–cell adhesion and endocytosis [60].

However, Arp2/3 does not initiate actin nucleation unless it is activated, in the presence of ATP, by a NPF. The class I NPFs and are the major regulators of Arp2/3-mediated actin nucleation [61]. Proteins like Wiskott–Aldrich syndrome protein (WASP) and junction-mediating and regulatory protein (JMY) are typical examples of class I NPFs which are defined by the presence of a WCA domain; which consists of a WH2 domain (W), a central, or connector, region (C), and an acidic region (A) (Fig. 3). The WCA region is the minimal sequence element that is required for potent activation of Arp2/3-mediated actin nucleation [55]. The WH2 domain allows binding and sequestering of actin monomers while the acidic region interacts with Arp2/3 (Fig. 3). In general, the N-terminal regions of NPFs are divergent and provide interaction sites for regulatory factors [61] (Fig. 3). It is particularly relevant that many NPFs are associated with the membrane and can therefore locally activate the Arp2/3 complex [62].

A second class of NPFs lack WH2 domains, but are able to interact with Arp2/3 and F-actin [61]. Cortactin, a class II NPF, does not contain a WH2 domain, but interacts with the Arp2/3 complex, F-actin and a variety of actin regulatory factors such as neuronal-WASP (N-WASP) [63]. Cortactin has been shown to regulate assembly of branched actin filaments by a variety of mechanisms including Arp2/3 activation and enhancing F-actin stability [63].

The formin proteins are a third class of actin nucleators which function independently of both the Arp2/3 complex and NPFs, resulting in unbranched actin filaments [64]. A fourth class of actin nucleators, the tandem-monomer-binding proteins, are comprised of proteins such as JMY and Spire [61, 62]. This group contains tandem G-actin binding motifs (typically WH2 domains; Fig. 3) that bring together actin monomers to form an actin nucleation seed resulting in unbranched actin fibres [65]. JMY is unique in that it can act as both a class I NPF (dependent on Arp2/3 binding for actin nucleation) while also able to nucleate actin in an Arp2/3-independent fashion (through its tandem G-actin binding motifs), resulting in both branched and unbranched actin filaments [66, 67].

Actin filaments are involved in many fundamental cellular processes ranging from membrane ruffling and cell motility to membrane trafficking and transport processes such as endocytosis [52, 54]. Given the fact that actin filaments can transport proteins and organelles as well as their intimate association with membranes, it is perhaps not surprising that recent work has begun to uncover roles for actin and actin nucleation proteins in autophagic processes.

## Actin in autophagosome formation

Early studies had demonstrated a role for the cytoskeleton, in particular the microtubule system, and molecular motors in various aspects of autophagy. For example, LC3 interacts with microtubules via the microtubule-associated proteins MAP1A, MAP1B or MAP1S [68, 69] and during starvation, studies have shown that early autophagosomal structures move along the microtubules [70]. Several early reports suggested a role for the actin cytoskeleton in autophagy [71, 72] although these relied on the use of actin-binding drugs which induce gross morphological changes to cells. As a result, it was unclear whether actin played a direct role in autophagosome formation or the subsequent morphological changes influenced cellular functions that impacted on autophagy.

### Yeast

Later, studies were able to demonstrate that in yeast, although actin filaments are dispensable for bulk autophagy, they are required for cargo selection for certain types of autophagy [73–75]. Specifically, Reggiori et al., demonstrated that in *Saccharomyces cerevisiae* (*S. cerevisiae*) the selective autophagy pathways cytoplasm to vacuole targeting (Cvt) and pexophagy (selective degradation of peroxisomes) were significantly impaired in cells grown in the presence of latrunculin A (a toxin that inhibits actin polymerisation) or particular actin mutants, whereas bulk autophagy was not [74]. Further they demonstrated that Atg9 cycling between the mitochondria and the phagophore assembly site was similarly affected and that the actin-dependent localisation of Atg11 at the phagophore assembly site was responsible for Atg9 cycling [74, 75]. Follow-up studies demonstrated selective autophagy required the Arp2/3 complex and that Arp9 cycling was dependent upon Arp2 and *arp2* mutant yeast cells displayed a block in selective, as well as a partial block in bulk, autophagy [76]. Interestingly, this group also demonstrated that the yeast Arp2/3 activators *las17* (N-WASP homologue) and *pan1* were necessary for selective autophagy [76].

### Mammalian cells

In mammalian cells up until recently there was very little known about the role of actin in autophagy. Aguilera et al., showed that de novo polymerisation of actin is necessary at certain steps during the autophagic process induced by starvation or rapamycin [77]. Inhibiting actin polymerisation with toxins (latrunculin B and cytochalasin B) prevented autophagosome maturation, but not fusion with the lysosome during basal and starvation induced autophagy [77, 78]. ATG14, DFCP1 and Beclin1 were shown to co-localise with actin during starvation induced autophagy, while LC3 co-localised with actin only sporadically, leading the authors to conclude that actin was involved in very early stages of autophagosome formation, likely at the omegasome [77]. Recent studies have shown that actin and LC3 co-localise under autophagy conditions [79, 80] and actin and LC3 co-localisation occurred at the isolation membrane, where actin could also co-localise with Atg5, Atg14, Atg16 and ULK1 [79].

Several recent reports have demonstrated a role for the NPFs JMY and WASP homologue associated with actin, membranes and microtubules (WHAMM) in autophagosome formation. JMY was originally identified as a p300 interacting protein and subsequently shown to influence transcription where it can act as a p53 co-factor [81, 82]. Follow-up work showed JMY to be a novel cytoplasmic actin nucleation promoting factor that re-localises to the nucleus under certain stressors such as anoxia or DNA damage [66, 67, 83]. Cytoplasmic JMY has been shown to influence cell motility and invasion and can localise to the lamellipodia [66, 67, 83]. JMY contains three WH2 domains and interestingly can nucleate actin in both an Arp2/3-dependent and independent fashion [66, 67]. JMY has also been shown to be localised to the ER and *trans*-Golgi network where it aids in vesicle trafficking [84]. We have shown that JMY localises to autophagosomes and through its ability to nucleate actin, can influence autophagosome formation [80]. JMY was shown to increase autophagosome number and size during both drug and starvation-induced autophagy to positively impact on cell survival [80]. Arp2/3-dependent actin nucleation was shown to play a role in autophagosome formation and JMY, via its WH2 domains, promoted LC3 and actin co-localisation at the autophagosome [80]. Interestingly, JMY can interact with LC3 via a LIR [47] in its N-terminal region and mutating this LIR resulted in lack of autophagosomal localisation of JMY as well as a lack of actin co-localisation [80]. This suggests the LIR directs JMY to autophagosomal membranes where it is activated to nucleate new actin filaments. Factors involved in the regulation of JMY nucleating activity have not yet been identified, but it would be interesting to explore the possibility that PI(3)P produced at the autophagosomal membrane may aid in the localised activation of JMY.

WHAMM was originally identified as an actin nucleator related to JMY, although the two are highly divergent at their N-terminal regions [85]. WHAMM contains two WH2 domains and can stimulate Arp2/3-dependent actin nucleation [85]. In addition, WHAMM was shown to bind microtubules and play a role in ER-Golgi transport [85]. Recently Kast and co-workers demonstrated that WHAMM plays a role in autophagosome biogenesis. WHAMM was shown to form puncta on the ER surface that were propelled by actin comet tails, promoting the movement of the ER-tethered membranes through an Arp2/3-mediated actin nucleation mechanism [86]. WHAMM puncta were found to co-localise with LC3 and DFCP1 but not the early pre-autophagosome marker Atg14 and WHAMM was found to positively influence autophagosome size and number [86]. Together the data support a role for WHAMM in autophagosome formation through an Arp2/3-dependent actin comet tail mechanism. Neither JMY nor WHAMM were seen to co-localise with the lysosomal marker LAMP1, suggesting that both proteins may be involved in earlier stages of autophagosome biogenesis [80, 86]. Whether JMY and WHAMM perform similar or overlapping roles is yet to be determined, but it is likely that the various actin nucleators may respond to different autophagy stimuli and/or play roles at different subcellular locations.

In addition, the WASP family protein WASH (an Arp2/3 activator) generates actin networks on endocytic vesicles and is important for sorting and maturation steps [87, 88]. More recently, WASH was shown to function in autophagic pathways in *Dictyostelium* where it is required to maintain lysosomal flux in a process requiring actin assembly [89, 90]. In mammalian cells WASH depletion has been shown to decrease autophagy and survival [91]. In contrast, WASH knockout mice exhibit early embryonic lethality and extensive autophagy, demonstrating in this situation an inhibitory effect of WASH on autophagy [92]. Mammalian WASH was found to interact with Beclin1 leading to an inhibition of Vps34 kinase activity, suggesting that WASH might influence an early stage of autophagosome formation [92]. These differences in outcome may be due to experimental methods (i.e., siRNA-mediated depletion versus knockout) or may be influenced by tissue or developmental differences, and further work is required to determine the exact role of WASH in mammalian autophagy. Moreover, the role of WASH-mediated actin nucleation in mammalian autophagy has not been fully explored, but it is tempting to speculate that actin nucleation is likely to play a role whose outcome could be both positive and negative dependent upon the context.

Atg9 is thought to be involved in the delivery of membranes to pre-autophagosome structures and autophagosomes [32, 33]. Trafficking of Atg9 from the plasma membrane to recycling endosomes via early endosome compartments is important for autophagosome biogenesis [31]. Recent work has demonstrated that actin is localised around Atg9A vesicles and this is involved in Atg9A trafficking [93]. Moreover, three actin nucleators, Annexin A2, Spire1 and Arp2 were shown to play a role in the actin-dependent Atg9A sorting. Actin was shown to be localised around Atg9A vesicles which was reduced upon Annexin A2 knockdown. Small molecule-mediated inhibition of Arp2 or Arp2 knockdown also decreased the localisation of actin around Atg9A vesicles as did perturbation of actin polymerisation, which in addition decreased Atg9A trafficking [93]. Together the data suggest that Annexin A2 via its effectors Arp2 and Spire1 regulate Arp9A trafficking and autophagosome formation via actin. Since Spire is a WH2 domain-containing tandem-monomer binding NPF [65], it will be of interest to explore the role of Spire’s actin nucleation activity in autophagosome formation.

One of the key questions in autophagosome formation is how the membrane shape is generated during the elongation and maturation of the developing autophagosome. Recent work has shed some light on this important question to demonstrate that during starvation the actin capping protein CapZ promotes actin nucleation inside the isolation membrane [79]. In CapZ-depleted cells, LC3 and DFCP1 (an omegasome marker protein [17]) positive omegasomes were found to exhibit an enlarged ring-shaped or tubular morphology, suggesting that autophagosome formation was impaired [79]. It is likely that PI(3)P (enriched within the omegasome due to Vps34 activation) stimulated actin polymerisation through dissociation of CapZ from actin fibres [79]. The actin-binding and filament-severing protein cofilin as well as the Arp2/3 complex protein p41-Arc were found inside the isolation membranes. Together their data led the authors to suggest that Arp2/3-dependent branched actin formation inside the forming omegasome provides the force for membrane curvature in the developing isolation membrane [79]. This hypothesis is supported by other experimental evidence demonstrating that actin fibres were found to be associated with the omegasome [77] and the fact that several groups have now demonstrated the Arp2/3-dependent actin nucleation is required for autophagosome formation (for example, [80, 86]).

## Actin in autophagosome to lysosome fusion

A number of proteins that can influence the actin cytoskeleton have been suggested to play a role in fusion of the lysosome with the autophagosome. Autophagosomes need to be transported to the lysosomes in order to fuse and thus the requirement for a structural component capable of providing a means of movement is perhaps not unexpected. While studies have shown that the microtubule system may be required for trafficking of autophagosomes, as well as formation, the mechanisms are not entirely clear [48, 94, 95]. It may be that cells utilise different transport systems dependent upon cell type and/or autophagy stimuli and these could affect various stages of autophagosome formation and maturation.

Interestingly, evidence suggests that actin nucleation may be required to mediate fusion of the autophagosome with the lysosome. Lee and co-workers have shown that during quality-control autophagy (disposal of damaged organelles and/or protein aggregates) histone deacetylase 6 (HDAC6)-mediated actin remodelling is required for fusion of autophagosomes to lysosomes [96]. Specifically, HDAC6 binds to ubiquitinated protein aggregates where it recruits cortactin-dependent actin remodelling machinery. Subsequently, F-actin formation at these sites enables fusion of the autophagosome with the lysosome and clearance of the aberrant aggregates [96]. Using an in vitro fusion assay this group was able to further demonstrate that autophagosomes purified from cells under starved conditions did not require HDAC6 or actin for fusion, in contrast to those from cells grown under normal growth conditions [96]. This is particularly striking and suggests that there are distinct differences in autophagosomes depending on the cellular conditions and further highlights the complexity in understanding the mechanistic details regarding autophagosome formation.

## Membrane regulation of actin nucleation

### Phosphoinositides

Actin has long been known to associate with membranes and plasma membrane associated phosphoinositides play a significant role in the regulation of actin nucleation [97]. For example, the actin capping protein CapZ is known to bind phosphatidylinositol-4,5-bisphosphate (PI(4,5)P) and PI(4)P to result in dissociation of Capz from the plus end of actin filaments, thus stimulating actin polymerisation at the barbed, fast growing, ends [98]. As previously discussed, PI(3)P formation at the omegasome is important in stimulating localised actin nucleation by displacing Capz from the barbed end of an existing actin filament [79]. A number of actin binding proteins can associate with phosphoinositides and in particular phosphoinositide binding by actin nucleators is important in activation of the WCA region (Fig. 3). For example, WASP is normally maintained in an inactive state in cells through intramolecular interactions which inhibit its WCA region [61]. Binding to PI(4,5)P_2_ as well as the small GTPase Cdc42 relieves this inhibition to facilitate Arp2/3-dependent actin nucleation [99]. It may be that similar to CapZ these proteins are able to interact with PI(3)P to influence their function. The actin nucleator Spire contains a FYVE domain in its C-terminal region known to be important for membrane targeting [100] (Fig. 3) and FYVE domains, found in proteins such as the early autophagy marker DFCP1, enable binding to PI(3)P [101]. It is likely that localised signals will influence actin nucleation at the autophagosomal membrane and similar to what has been shown to occur at other membrane surfaces this could be driven by membrane-derived phosphoinositides. It could be that the localised pool of PI(3)P created through Vps34 activation may, in turn, drive activation of nucleation promoting factors such as JMY, helping in the maturation and expansion of the autophagosome.

### Actin binding proteins

Another way in which actin is known to be regulated at membranes is through the membrane association of small GTPases and other actin regulatory factors [97]. Most actin regulatory proteins require activation and the small GTPases of the Rho superfamily are well-known modulators of actin filament assembly via GTPase signalling cascades [102]. As described, Cdc42 is involved in WASP signalling [99], and binding of the Rho family member Rac to the WASP family member WASP family Verprolin‐homologous (WAVE) is involved in its activation [103]. In general, binding to these membrane-bound small GTPases helps to relieve the inhibited state and leads to Arp2/3-dependent actin nucleation. Interestingly, members of the Rho family of GTPases have been shown to play a role in autophagy. For example, Aguilera et al., have shown that the actin regulatory protein RhoA is involved in autophagosome formation during starvation [77]. Cells over-expressing active RhoA had increased autophagosome numbers during starvation-induced autophagy while cells with reduced RhoA (either siRNA-mediated or toxin inactivation) exhibited reduced autophagosome numbers [77]. Although currently unexplored, it is tempting to speculate that RhoA-mediated actin dynamics may be involved in autophagosome formation. Rho kinase (ROCK) is a family of serine/threonine kinases (ROCK1 and 2) that are important effectors of Rho GTPases [104]. Interestingly, Beclin1 has been identified as a binding partner and substrate for ROCK1 and activation of ROCK1 was shown to promote autophagy and phosphorylation of Beclin1 [105]. In support of this another study has shown that when ROCK activity was inhibited chemically or reduced by siRNA-mediated depletion in HeLa cells a decreased number of autophagosomes were observed during starvation [77]. Although no effect of chemically inhibiting ROCK activity in the presence of a constitutively active RhoA was observed, suggesting that other downstream effectors may be involved [77]. In contrast, in HEK 293 cells ROCK1 activation inhibited autophagy while the opposite was true for ROCK inhibition during starvation induced autophagy [106]. Again, while it is currently unknown if actin dynamics are involved in these effects, there may be isoform or cell type specific differences that play a role in determining the factors involved and their outcomes. The Rac family, comprising of Rac1, Rac2 and Rac3, are small GTPase proteins with roles in a number of cellular processes including cytoskeletal reorganisation [107]. Interestingly, Rac1 has been shown to inhibit starvation-induced autophagy [77] while Rac3, but not Rac1 or Rac2, inhibited basal autophagy [108] again suggesting that different actin regulatory proteins may have positive or negative roles dependent upon cell type and/or autophagy stimuli. Further work will be required to explore this possibility and determine if these effects are dependent on actin dynamics. Moreover, so far no reports have suggested these GTPases are found at autophagosomal membranes, although it is an interesting idea that is worth exploring.

Bin/amphiphysin/Rvs (BAR) domain proteins sense and participate in membrane binding [109] and are involved in a variety of cellular processes that require membrane remodelling [110]. BAR domain proteins also contain a variety of protein interaction domains and can thus function as scaffolding proteins. For example, BAR domain proteins can interact with Rho GTPases as well as actin-regulating proteins such as N-WASP and can thus influence localised actin nucleation at the membrane [111]. It is worth mentioning that in plants, the BAR domain protein SH3P2 binds PI(3)P and LC3 and promotes autophagosome formation [112], while in mammalian cells, the BAR domain proteins SNX18 and Bif-1 have been shown to promote autophagosome formation [113, 114]. While thus far unexplored, it may be that during autophagy, BAR domain proteins could sense the crescent-shaped omegasome at the earliest stages of autophagosome formation and recruit actin modulating proteins to the developing autophagosome.

## Actin-based motor proteins

Actin-based motor proteins such as myosins move along actin filaments dependent on the hydrolysis of ATP [115]. These motor proteins are involved in the movement of various cargos, including membrane-derived organelles, to appropriate cellular locations [115]. The first link between myosin and autophagy was from Tang and co-workers who demonstrated that overexpressing Atg1 in *Drosophila* caused the reorganisation of F-actin and induced myosin II activation due to increased phosphorylation of the myosin II regulatory light chain (MLC) by the kinase spaghetti squash activator (SQA) [116]. In mammalian cells ULK1 and the homologue of SQA, zipper-interacting protein kinase (ZIPK) were shown to interact, and increased MLC phosphorylation was observed during starvation. Moreover, siRNA-mediated depletion of ULK1 or ZIPK reduced myosin II activation, and ZIPK depletion reduced the size and number of autophagosomes during starvation [116]. In particular, myosin II activation was shown to be required for starvation-induced autophagy in *Drosophila* and MCF-7 cells where it was found to regulate Atg9 trafficking [116]. Under nutrient-rich conditions Atg9 is mainly located in juxta-nuclear regions where it co-localises with the *trans*-Golgi marker TGN46 and during starvation Atg9 is re-localised to late endosomes and autophagosomes in a myosin II-dependent fashion [116].

Other, non-conventional myosins have also been shown to have a potential role in autophagy. For example, MYO1C (a class I myosin) is an important regulator of lipid raft trafficking from intracellular storage compartments to the plasma membrane [117] and in cells depleted of functional MYO1C (using either siRNA or chemical inhibition) autophagosome–lysosome fusion was defective [118]. Myosin VI, in contrast to most other myosins, moves towards the minus end of actin filaments [119]. Studies have shown that myosin VI interacts with three proteins involved in cargo-selective autophagy; nuclear dot protein 52 (NDP52), optineurin, and TRAF6-binding protein (T6BP). These proteins target ubiquitinated cargo for autophagy-dependent degradation facilitated by myosin VI [120]. Tom1 was additionally identified as a myosin VI binding partner on endosomes and both Tom1 and myosin VI were required for fusion of the autophagosome with the lysosome [120]. During pathogen infection autophagy plays a key role through clearance of the pathogen in a process known as xenophagy [14] and NDP52 and myosin VI have been shown to promote autophagosome maturation during bacterial infection [121]. Recently, Tumbarello and co-workers also demonstrated that during bacterial infection, both myosin VI and its interactor TAX1BP1 are required for xenophagy [122].

While these studies did not specifically address the role of actin, the fact that myosins rely on actin interactions for their function strongly suggests the possible involvement of actin dynamics. It is likely that distinct myosins may play different roles in autophagic pathways and uncovering the link between these molecular motors and the impact of actin in their role will undoubtedly provide important insights into autophagosome formation.

## Conclusions and future perspectives

Autophagy is an essential process that is involved in normal cellular homeostasis, development as well as cell survival. Its influence over cell survival also means that autophagy is involved in numerous cellular stresses, such as hypoxia and the DNA damage response, where it can influence cell fate. Autophagy has thus been implicated in a variety of disease and pathological processes such as tumourigenesis, neurodegeneration and inflammation and as such is an increasingly important therapeutic target [1]. The newly discovered roles of actin nucleation in autophagy adds to the understanding of the autophagy process and opens up exciting new avenues for future research to uncover the molecular details involved.

Most autophagy research to date has focused on macroautophagy, the bulk turnover of cytosolic components. It is likely that actin dynamics and thus the regulatory proteins involved will differ dependent upon the form of autophagy. In particular, mitophagy (mitochondrial autophagy) may be a type of autophagy where actin nucleation could play a role. Mitochondrial movement relies on the cytoskeleton and dependent on the cellular context actin as well as motor proteins can influence mitochondrial motility [123]. Actin dynamics are also known to be important in mitochondrial division where actin nucleation provides the force required to split a mitochondrion during the fission process [124]. Of interest, a recent study has demonstrated that an isoform of the actin nucleator Spire (Spire1C) is localised to mitochondria where it plays a role in mitochondrial division [125]. Whether actin nucleating proteins like Spire play a role in mitophagy is yet to be determined, but given the important role of actin in mitochondrial dynamics it is a tantalising possibility. Additionally, although mTOR activity is a critical regulator of autophagy, a variety of signals feed into regulation of the mTORC1 complex via different effector pathways (Fig. 2). These diverse effector pathways could, in turn, determine what actin nucleating activity is recruited to the autophagosome. Moreover, various stimuli may result in autophagy via different mechanisms from the canonical autophagic pathway [126]. This would suggest that more in-depth analyses of the types of signalling mechanisms that are involved in autophagy induction and formation will help to uncover how different actin nucleators could provide specificity.

Furthermore, motor proteins such as myosins bind to, and travel along, actin filaments [127] and this could provide additional means by which actin could influence autophagy and the formation of the autophagosome. As described, myosins have been shown to influence autophagosome formation. Of note, MLC is a downstream target of ROCK [104], and although ULK1 activity has been shown to activate myosin II via MLC phosphorylation by SQA/ZIPK [116], perhaps ROCK provides further opportunity for links between ULK1 activity and MLC phosphorylation in a cell and context-specific fashion.

In yeast, recent work has demonstrated that Atg11 can activate Atg1 during selective autophagy [128] and Atg11 has been shown to track along actin cables to the phagophore assembly site [75]. It will be interesting to see if actin can influence Atg1/ULK1 activity and more generally if this applies to mammalian cells. The mammalian counterpart to yeast Atg11 is unclear, but FIP200 has some sequence similarity and has been proposed to be a mammalian hybrid homologue of yeast Atg11 and Atg17 [6]. FIP200 was originally identified as a focal adhesion protein that can interact with actin-binding proteins such as β-catenin and focal adhesion kinase [129, 130] and so it is perhaps likely that it may functionally link to actin.

An important consideration is what crosstalk might exist between the microtubule and the actin filament networks. Both systems have been implicated in autophagic processes under a variety of conditions (for a review on the role of microtubules in autophagy see [131]). Moreover, an interaction between actin filaments and microtubules is a widespread phenomenon that is involved in regulating fundamental processes such as cell migration [132]. The actin and microtubule networks can connect during, for example, the recycling of endosomes (see for e.g., [133]). Importantly, microtubule-associated proteins (MAPs) are central in facilitating interactions between microtubules and actin [134]. Since LC3 is a MAP binding protein [68], it is likely that a functional interaction between actin and microtubules, perhaps facilitated by LC3 and LC3 interacting proteins, occurs during autophagy. Additionally, NPFs may functionally link actin and microtubules during autophagy. The NPF WHAMM has been shown to bind to microtubules through a central coiled-coil domain [85] and while the role of microtubule function in JMY’s activities has not yet been explored, the fact that it too contains a central coiled-coil domain suggests that microtubule binding is a possibility (Fig. 3). It will be important to explore what effects these two systems might exert on autophagosome formation.

A novel recent study has shown that nuclear LC3 links degradation of nuclear lamina to autophagy during oncogene-induced senescence in which autophagy is upregulated [135]. This is thought to occur through a direct nuclear interaction between LC3 with lamin B1, facilitated by LC3 lipidation which could function to restrict cell proliferation [135]. Additionally, starvation-induced nuclear autophagy has been described in yeast [136]. What role nuclear actin may play in this process has not been investigated, but NPFs such as JMY can shuttle to the nucleus dependent upon the cellular conditions [66, 83, 137]. Whether NPFs and actin play a role in any nuclear aspects of autophagic clearance is an interesting and unique possibility that remains to be explored.

The recently described roles for NPFs in the autophagic process may provide an avenue for specificity dependent on the organism, location and/or type of stimuli. While NPFs share common C-terminal domains important in actin nucleation and Arp2/3 complex activation they are often highly divergent at their N-termini (Fig. 3). In addition, actin-nucleating proteins are localised to distinct cellular locations and organelles. For example, mammalian WASH localises to early and recycling endosomes [87, 88]. JMY has been shown to localise to the ER and *trans*-Golgi network where it aids in vesicle trafficking [84] and WHAMM has been shown to localise to specific ER sites [86] where it is involved in ER to Golgi transport [85]. It is possible that actin-nucleating proteins could play distinct roles at different steps in the autophagic process based on their unique localisation. The divergent N-terminal region, which may be involved in localisation, would also allow for distinct modes of activation; providing temporal as well as spatial control over actin nucleating activity.

**Fig. 4:**
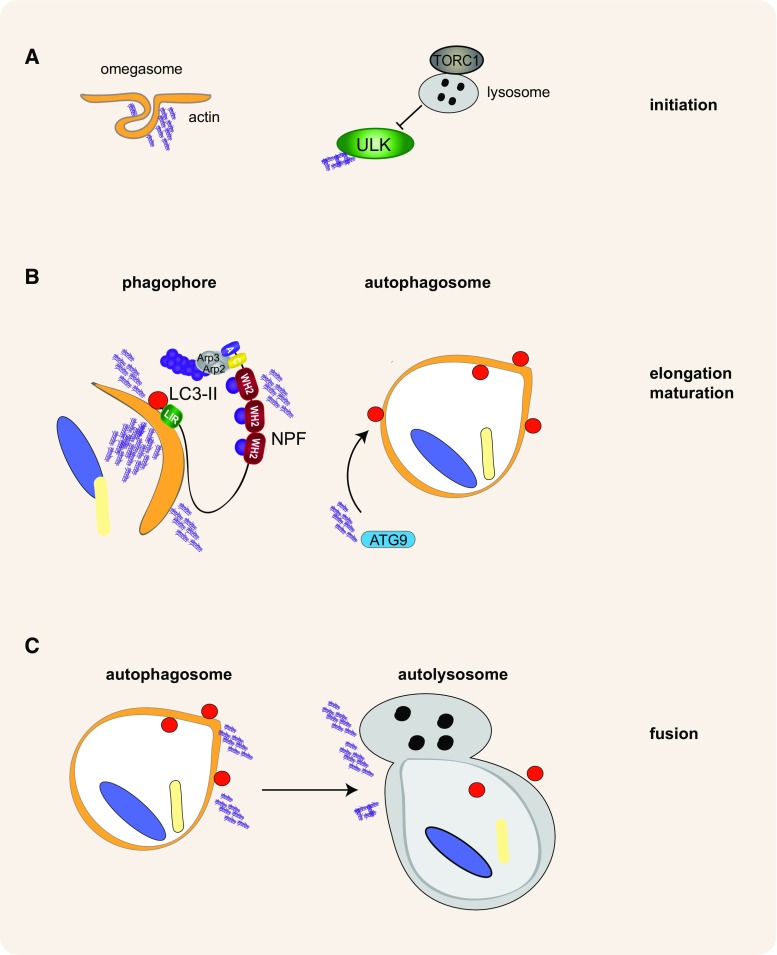
Hypothetical roles of actin in autophagosome formation. Actin nucleation could play a variety of roles directed by different actin regulatory proteins at different stages of autophagosome formation. **a** During the initial stages of autophagosome formation, actin may be nucleated at the developing omegasome, helping to shape and develop the pre-autophagosomal membrane. In addition, actin nucleation may be involved in regulating ULK complex activity and/or recruitment to the initiation site. **b** During elongation and maturation actin nucleation can help shape the developing phagophore; moreover, actin nucleating proteins may be recruited in order to facilitate actin nucleation and/or aid in the recruitment of Atg9 or membrane sources to the developing phagophore. **c** Actin nucleation is thought to play a role in fusion of the autophagosome with the lysosome. This may involve trafficking of the developing phagophore to the lysosome (or vice versa) in addition actin may be linked to Rab/SNARE activity and aid directly in the fusion process

Currently it is unclear precisely how actin functions during autophagy, but it appears likely that actin regulates multiple steps along the way to creation of an autophagosome; from isolation membrane curvature to expansion of the maturing autophagosome leading ultimately to its fusion with the lysosome (Fig. 4). Future studies to uncover the varied roles of actin and actin nucleation in autophagosome formation will undoubtedly uncover new avenues for modulating cell fate during autophagy, an important area of research in terms of human health and disease.

## Abbreviations

AMBRA1: Autophagy/beclin-1 regulator 1
AMPK: AMP-activated protein kinase
Arp2/3: Actin-related protein 2/3
Atg: Autophagy-related gene
BAR: Bin/amphiphysin/Rvs
DFCP1: Double FYVE domain-containing protein 1
ER: Endoplasmic reticulum
FIP200: Focal adhesion kinase family-interacting protein 200 kDa
GABARAPs: γ-Aminobutyric acid receptor-associated proteins
HDAC6: Histone deacetylase 6
JMY: Junction-mediating and regulatory protein
LC3: Atg8/microtubule-associated protein 1A/B light chain 3
LIR: LC3-interacting region
MAPs: Microtubule-associated proteins
mTORC1: Mammalian target of rapamycin complex 1
NDP52: Nuclear dot protein 52
NPF: Nucleation promoting factor
PE: Phosphatidylethanolamine
pan1: Poly (A) nuclease 1
PI(3)K: Phosphatidylinositol-3-kinase
PI(3)P: Phosphatidylinositol-3-phosphate
PI(4)KIIα: Phosphatidylinositol-4-kinase IIα
PI(4)P: Phosphatidylinositol-4-phosphate
PI(4,5)P_2_: Phosphatidylinositol-4,5-bisphosphate
RAPTOR: Regulatory-associated protein of mTOR
SQA: Spaghetti squash activator
T6BP: TRAF6-binding protein
TAX1BP1: TAX1-binding protein 1
TSC1/2: Tuberous sclerosis protein 1 and 2
ULK: Unc-51-like kinases
UVRAG: UV radiation resistance-associated gene protein
Vps34: Vacuolar protein sorting 34
WAVE: WASP family verprolin‐homologous
WASP: Wiskott–Aldrich syndrome protein
WH2: WASP-homology 2
WHAMM: WASP homologue associated with actin, membranes and microtubules
WIPI: WD-repeat domain phosphoinositide-interacting protein
ZIPK: Zipper-interacting protein kinase
